# Dataset on the distribution location and biological traits of freshwater fishes in the Yangtze River Basin

**DOI:** 10.1016/j.dib.2018.10.093

**Published:** 2018-10-26

**Authors:** Bin Kang, Xiaoxia Huang, Yunzhi Yan, Yunrong Yan, Hungdu Lin

**Affiliations:** a College of Fisheries, Ocean University of China, Qingdao 266003, China; b Key Laboratory of Atmospheric Environment and Processes in the Boundary Layer Over the Low-latitude Plateau Region, School of Earth Science, Yunnan University, Kunming 650091, China; c College of Life Sciences, Anhui Normal University, Wuhu 241002, China; d College of Fisheries, Guangdong Ocean University, Zhanjiang 524088, China

## Abstract

In this data article, we provide the scientific and theoretical data on fish taxonomy including class, order, family, and genus in the Yangtze River. The Yangtze basin is divided into 56 units, and their geological information including latitude, longitude, latitude, and channel length is recorded. Fish presence/absence data at the unit scale are reported. Biological traits including morphological, physiological, and ecological characters of each fish species are also described, numeralized, and reported. These data are the foundation of the analyses and results in the article “Continental-scale analysis of taxonomic and functional fish diversity in the Yangtze River” (Kang et al., 2018).

**Specifications table**

**Table:** 

Subject area	Biology
More specific subject area	Biodiversity and conservation
Type of data	Table, Excel
How data was acquired	Collection and revision of available literatures, Surveys, GIS
Data format	Raw
Experimental factors	Brief description of any pretreatment of samples
Experimental features	Very brief experimental description
Data source location	The Yangtze Basin
Data accessibility	Data are with this article

**Value of the data**

• Fish lists of the Yangtze fishes were collected from available materials and revised according to the latest version of Fishbase for taxonomic criterion.
• Fish distribution data can be incorporated into other biogeographical studies concerning the environmental pollution, climate change, and human activities.
• Surveyed biological traits of fishes in the whole basin can provide insights in species interaction, ecosystem function, and conservational decision-making.

## 1 Data

The Yangtze River, covering multiple types of landforms and climatic zones, supports abundant fish diversity and resources. In this data article, based on the natural river system and discharge, we divided the basin into 56 units [2] and extracted their geological data (Table 1, see Figure 1 in Ref. [1]), including longitude, latitude, altitude, and channel length of each unit. On the basis of available literatures, we reviewed and updated the freshwater fish species and their distribution at unit scale (Appendix A) to determine the species richness. According to their phylum (class, order, family, and genus; Table S2), taxonomic diversity of each unit was calculated. Biological traits were used to determine functional diversity. The data catalogs include body shape, feeding habit, trophic level, water zone, water column position, and water temperature (Table 2), and the details of each species were determined (Appendix B). We quantified the dissimilarities of species richness, taxonomic, and functional diversity among all the units, and traced the process of species turnover and nestedness.

**Table 1:** Units of the Yangtze Basin and corresponding geological information

ID	Channel length (km)	Altitude (m)		Longitude		Latitude	
		Minimum	Maximum	Minimum	Maximum	Minimum	Maximum
1	24227	3336	6384	90.5478	97.7753	32.4282	35.7737
2	4046	2236	5328	97.3401	99.1676	28.7725	32.5481
3	8101	1493	5835	97.2879	100.7906	27.7440	32.9908
4	4775	1345	5234	98.9463	100.6859	26.6235	28.9495
5	14736	267	4137	100.0511	104.9484	24.4736	28.6795
6	5576	257	3935	100.3812	104.6528	25.9785	28.8958
7	24355	984	5658	96.8339	102.7022	26.6163	34.2220
8	13568	359	6243	99.6284	103.7501	28.2261	33.6468
9	2554	366	4887	102.2893	103.7487	29.3925	30.9851
10	9557	266	5373	102.6089	104.6774	28.4292	33.1480
11	5541	228	4473	103.6824	105.8272	28.8312	31.7099
12	8580	488	4439	102.5303	107.0305	32.5766	34.5264
13	7531	188	5082	103.7457	106.2714	29.2820	33.1203
14	5810	161	3716	104.5294	107.0649	29.4557	32.9111
15	7442	196	2532	106.2286	109.0098	29.9961	32.7647
16	7151	50	2960	104.4653	111.4596	28.7153	31.7256
17	7030	48	2115	104.3225	111.1789	27.8508	31.0717
18	2954	191	2182	104.6273	106.9917	27.2400	28.9176
19	2268	868	2782	104.3138	106.3228	26.1546	27.5326
20	5386	289	2334	105.8762	108.8045	26.0785	28.5762
21	2081	207	1880	107.3738	109.3705	28.0461	30.1985
22	2323	136	2182	106.8689	108.9405	28.0002	30.2347
23	18983	116	3475	106.1247	111.7460	31.4140	34.1890
24	6829	61	2002	111.6067	113.6475	31.6484	33.7411
25	12364	8	2812	110.3243	114.2781	30.1085	32.6972
26	2819	22	2168	108.5549	111.4458	29.6591	30.9337
27	2388	21	1753	110.9567	112.2133	30.2726	31.7119
28	5717	−46	436	111.9865	114.0741	29.4247	31.0669
29	3986	16	900	112.7206	114.3730	30.6019	32.3190
30	5260	−11	1584	114.2417	116.0750	29.8482	31.6399
31	7129	−35	1432	113.1247	116.0318	29.0368	30.6721
32	3006	18	1932	109.7017	111.9410	29.0227	30.1394
33	3345	206	2099	107.2797	109.5991	26.0607	27.2583
34	2242	164	1907	109.0253	110.5648	25.9743	27.2043
35	5112	103	2416	107.3883	110.8973	26.8547	28.2982
36	7901	28	1803	108.7045	111.8334	27.8699	30.0478
37	3001	169	1991	110.2306	111.8175	25.8305	27.7907
38	2350	27	1522	110.7214	112.3855	27.6686	28.7645
39	9561	48	1885	110.5363	114.0491	24.6393	27.2146
40	4180	24	1203	111.4690	113.1299	26.4350	28.4770
41	5497	19	1967	112.7549	114.2699	25.9564	28.6459
42	1506	31	1536	113.0758	114.1594	28.4385	29.4870
43	10688	9	1355	110.9241	113.2804	28.3440	30.4191
44	7961	79	1673	113.4955	116.6291	24.4971	27.1595
45	5586	29	2001	113.8132	116.0289	25.9200	27.6499
46	4984	8	1680	113.9645	116.1365	27.4435	28.7701
47	3694	19	1411	115.5999	117.1689	26.5044	28.3164
48	2792	19	2088	116.5294	118.6071	27.5402	28.9761
49	2342	16	1556	116.9279	118.2313	28.5216	30.0456
50	2981	15	1671	113.9876	115.8174	28.3687	29.5094
51	6259	−1	1359	115.4142	117.1315	28.2248	29.9156
52	13228	−112	1610	115.4292	119.6564	29.7055	32.7349
53	8589	−21	1716	116.1911	119.6600	29.6024	32.2603
54	5266	−7	16	119.6564	121.9232	31.6905	32.6533
55	284	−21	11	121.0622	122.2725	30.9432	31.8860
56	15671	−28	1459	119.1162	121.9261	30.0533	32.3964

**Table 2:** Catalogs of fish species traits and detailed description for determining functional diversity.

Category	Trait	Description	Type
Morphology	Body length	Related with growth rate, as well as mortality rates, longevity and reproduction	continuous numerical
	Body depth	The vertical distance at the top of the body	
	Head depth	The head depth along the eye׳s vertical axis	
	Head length	The vertical distance from the snout to the posterior margin of operculum	
	Pectoral fin length	Distance from starting point to end of the pectoral fin	
	Distance between insertion of the pectoral fin to Bottom of the body (PFi)	PFi/PFb, pectoral fin position, representing maneuverability of the pectoral fin	
	Body depth at the level of the pectoral fin Insertion (PFb)		
	Caudal fin length (CFd)	CFd/CPd, the caudal peduncle throttling, representing caudal propulsion efficiency through reduction of drag	
	Caudal peduncle minimal depth (CPd)		
	Eye size (Ed)	The horizontal maximum distance between the anterior and posterior border of the eye	
	Distance between centre of the eye to bottom of the head (Eh)	Eh/Hd, representing the eye position	
	Distance from top of the mouth to bottom of the head (Mo)	Mo/Hd, oral gape position, representing feeding method in the column	
Trophic adaptability	Feeding habit	Parasitic	1
		Detritivorous	2
		Planktivorous	3
		Herbivorous	4
		Omnivorous	5
		Invertivorous	6
		Carnivorous	7
		Piscivorous	8
	Trophic level	The position a species occupies in a food chain	numerical
Habitat	Water column position, the favorite water layer for species living	Pelagic	1
		Pelagic-neritic	2
		Benthopelagic	3
		Demersal	4
	Water zone, different places with species appearance	Marine	1
		Marine, river, lake	2
		Marine, river	3
		River	4
		River, Lake,	5
		River, Lake, stream	6
		River, stream	7
		Lake	8
		Lake, stream	9
		Stream	10
	Water temperature, the suitable temperature for species living	Tropical	1
		Subtropical	2
		Temperate	3
		High altitude	4

## 2 Experimental design, materials, and methods

The Yangtze Basin was dived into geographic 56 units (sub-basins) according to the natural river system and discharge, each unit with annual discharge larger than 300 × 10^8^ m^2^. Then the maximum, minimum, and mean values of longitude, latitude, and altitude were extracted; the channel length of each unit was calculated.

To clarify the fish fauna in the basin, we collected available literatures including monographs, published papers, investigative reports, and additional records. The compiled data were revised following Fishbase [3] to avoid invalid species, as well as synonyms and homonyms.

At the unit scale, the records of species locality were identified for constructing the species distributional data matrix. The presence/absence data were scored ‘1’ for the presence of a species in a unit, and ‘0’ for its absence. An aggravate data matrix on species taxonomy was compiled with order, family, and genus as columns, and species as rows.

We constructed a functional traits matrix regarding morphological, physiological, and ecological characters. Morphological parameters (the shape of body, head, eye, fin, etc.) were directly measured from available formalin fixed specimens. We extracted data for feeding habit and trophic level from the literature [4] and Appendix A. Data on water zone, water column position, and water temperature suitable for a species were extracted by reviewing the distributional information from referential sampling reports. When physiological and ecological knowledge of a species was not available, we extrapolated the data for the genus to the species level. Traits in ordinal, nominal, and continuous data were then numeralized: For the numerical data, an average value was calculated and assigned to each individual [5] when more than one value was available for a given species; for the nominal data, the trait status received different values based on its category.
