# Supplementary material for: Dataset on the distribution location and biological traits of freshwater fishes in the Yangtze River Basin
Source: Data Brief. 2018 Oct 26;21:1066–70. doi: 10.1016/j.dib.2018.10.093 (PMC6226585; doi:10.1016/j.dib.2018.10.093)
Supplement: Supplementary file 1 — Supplementary material [file mmc1.docx]

AUTHOR DECLARATION

We wish to confirm that there are no known conflicts of interest associated with this publication and there has been no significant financial support for this work that could have influenced its outcome.

We confirm that the manuscript has been read and approved by all named authors and that there are no other persons who satisfied the criteria for authorship but are not listed. We further confirm that the order of authors listed in the manuscript has been approved by all of us.

We confirm that we have given due consideration to the protection of intellectual property associated with this work and that there are no impediments to publication, including the timing of publication, with respect to intellectual property. In so doing we confirm that we have followed the regulations of our institutions concerning intellectual property.

We understand that the Corresponding Author is the sole contact for the Editorial process (including Editorial Manager and direct communications with the office). He is responsible for communicating with the other authors about progress, submissions of revisions and final approval of proofs. We confirm that we have provided a current, correct email address which is accessible by the Corresponding Author and which has been configured to accept email from ([kangbin@ouc.edu.cn](mailto:kangbin@ouc.edu.cn)).

Signed by all authors as follows:

Bin Kang, 2018-10-19

Xiaoxia Huang, 2018-10-19

Yunzhi Yan, 2018-10-19

Yunrong Yan, 2018-10-19

Hungdu Lin, 2018-10-19
