# Supplementary material for: Dataset on the distribution location and biological traits of freshwater fishes in the Yangtze River Basin
Source: Data Brief. 2018 Oct 26;21:1066–70. doi: 10.1016/j.dib.2018.10.093 (PMC6226585; doi:10.1016/j.dib.2018.10.093)
Supplement: Supplementary file 4 — Supplementary material [file mmc4.doc]

Table 2. Taxonomic list of the Yangtze freshwater fishes.

| Order | Family | Genus | Species |
| --- | --- | --- | --- |
| Acipenseriformes | Acipenseridae | *Acipenser* | *Acipenser dabryanus* |
|  |  |  | *Acipenser sinensis* |
|  | Polyodontidae | *Psephurus* | *Psephurus gladius* |
| Clupeiformes | Clupeidae | *Tenualosa* | *Tenualosa reevesii* |
|  | Engraulidae | *Coilia* | *Coilia brachygnathus* |
|  |  |  | *Coilia mystus* |
|  |  |  | *Coilia nasus* |
| Salmoniformes | Salmonidae | *Brachymystax* | *Brachymystax lenok* |
|  |  | *Hucho* | *Hucho bleekeri* |
|  |  | *Hemisalanx* | *Hemisalanx brachyrostralis* |
|  |  | *Neosalanx* | *Neosalanx oligodontis* |
|  |  |  | *Neosalanx taihuensis* |
|  |  | *Salanx* | *Salanx chinensis* |
|  |  |  | *Salanx prognathus* |
| Anguilliformes | Anguillidae | *Anguilla* | *Anguilla japonica* |
| Cypriniformes | Catostomidae | *Myxocyprinus* | *Myxocyprinus asiaticus* |
|  | Cyprinidae | *Aphyocypris* | *Aphyocypris chinensis* |
|  |  | *Atrilinea* | *Atrilinea macrolepis* |
|  |  | *Ochetobius* | *Ochetobius elongatus* |
|  |  | *Opsariichthys* | *Opsariichthys bidens* |
|  |  | *Zacco* | *Zacco chengtui* |
|  |  |  | *Zacco platypus* |
|  |  | *Rhynchocypris* | *Rhynchocypris lagowskii* |
|  |  |  | *Rhynchocypris oxycephalus* |
|  |  | *Anabarilius* | *Anabarilius liui liui* |
|  |  |  | *Anabarilius qionghaiensis* |
|  |  |  | *Anabarilius songmingensis* |
|  |  |  | *Anabarilius xundianensis* |
|  |  | *Ancherythroculter* | *Ancherythroculter kurematsui* |
|  |  |  | *Ancherythroculter nigrocauda* |
|  |  |  | *Ancherythroculter wangi* |
|  |  | *Chanodichthys* | *Chanodichthys dabryi* |
|  |  |  | *Chanodichthys erythropterus* |
|  |  |  | *Chanodichthys mongolicus* |
|  |  |  | *Chanodichthys oxycephalus* |
|  |  | *Culter* | *Culter alburnus* |
|  |  |  | *Culter oxycephaloides* |
|  |  | *Hemiculter* | *Hemiculter bleekeri* |
|  |  |  | *Hemiculter leucisculus* |
|  |  |  | *Hemiculter tchangi* |
|  |  | *Megalobrama* | *Megalobrama amblycephala* |
|  |  |  | *Megalobrama pellegrini* |
|  |  |  | *Megalobrama mantschuricus* |
|  |  |  | *Megalobrama terminalis* |
|  |  | *Parabramis* | *Parabramis pekinensis* |
|  |  | *Pseudohemiculter* | *Pseudohemiculter dispar* |
|  |  |  | *Pseudohemiculter hainanensis* |
|  |  |  | *Pseudohemiculter kweichowensis* |
|  |  | *Pseudolaubuca* | *Pseudolaubuca engraulis* |
|  |  |  | *Pseudolaubuca sinensis* |
|  |  | *Sinibrama* | *Sinibrama macrops* |
|  |  |  | *Sinibrama taeniatus* |
|  |  |  | *Sinibrama wui* |
|  |  | *Toxabramis* | *Toxabramis swinhonis* |
|  |  | *Distoechodon* | *Distoechodon tumirostris* |
|  |  | *Hypophthalmichthys* | *Hypophthalmichthys molitrix* |
|  |  |  | *Hypophthalmichthys nobilis* |
|  |  | *Plagiognathops* | *Plagiognathops microlepis* |
|  |  | *Pseudobrama* | *Pseudobrama simoni* |
|  |  | *Xenocypris* | *Xenocypris davidi* |
|  |  |  | *Xenocypris fangi* |
|  |  |  | *Xenocypris hupeinensis* |
|  |  |  | *Xenocypris macrolepis* |
|  |  |  | *Xenocypris yunnanensis* |
|  |  | *Acanthorhodeus* | *Acanthorhodeus chankaensis* |
|  |  | *Acheilognathus* | *Acheilognathus barbatulus* |
|  |  |  | *Acheilognathus barbatus* |
|  |  |  | *Acheilognathus elongatus* |
|  |  |  | *Acheilognathus gracilis* |
|  |  |  | *Acheilognathus hypselonotus* |
|  |  |  | *Acheilognathus imberbis* |
|  |  |  | *Acheilognathus macropterus* |
|  |  |  | *Acheilognathus meridianus* |
|  |  |  | *Acheilognathus omeiensis* |
|  |  |  | *Acheilognathus peihoensis* |
|  |  |  | *Acheilognathus polylepis* |
|  |  |  | *Acheilognathus tabira* |
|  |  |  | *Acheilognathus taenianalis* |
|  |  |  | *Acheilognathus tonkinensis* |
|  |  | *Rhodeus* | *Rhodeus fangi* |
|  |  |  | *Rhodeus lighti* |
|  |  |  | *Rhodeus ocellatus* |
|  |  |  | *Rhodeus sinensis* |
|  |  | *Tanakia* | *Tanakia himantegus* |
|  |  | *Acrossocheilus* | *Acrossocheilus hemispinus* |
|  |  |  | *Acrossocheilus parallens* |
|  |  |  | *Acrossocheilus fasciatus* |
|  |  |  | *Acrossocheilus jishouensis* |
|  |  |  | *Acrossocheilus Kreyenbergii* |
|  |  |  | *Acrossocheilus monticola* |
|  |  |  | *Acrossocheilus paradoxus* |
|  |  |  | *Acrossocheilus yunnanensis* |
|  |  | *Schizopygopsis* | *Schizopygopsis thermalis* |
|  |  | *Schizothorax* | *Schizothorax cryptolepis* |
|  |  |  | *Schizothorax heterochilus* |
|  |  |  | *Schizothorax chongi* |
|  |  |  | *Schizothorax davidi* |
|  |  |  | *Schizothorax dolichonema* |
|  |  |  | *Schizothorax grahami* |
|  |  |  | *Schizothorax griseus* |
|  |  |  | *Schizothorax kozlovi* |
|  |  |  | *Schizothorax labrosus* |
|  |  |  | *Schizothorax lissolabiatus* |
|  |  |  | *Schizothorax longibarbus* |
|  |  |  | *Schizothorax microstomus* |
|  |  |  | *Schizothorax ninglangensis* |
|  |  |  | *Schizothorax parvus* |
|  |  |  | *Schizothorax prenanti* |
|  |  |  | *Schizothorax sinensis* |
|  |  |  | *Schizothorax wangchiachii* |
|  |  |  | *Schizothorax yunnanensis* |
|  |  | *Sinocyclocheilus* | *Sinocyclocheilus grahami* |
|  |  |  | *Sinocyclocheilus multipunctatus* |
|  |  | *Bangana* | *Bangana rendahli* |
|  |  |  | *Bangana tungting* |
|  |  | *Discogobio* | *Discogobio tetrabarbatus* |
|  |  |  | *Discogobio yunnanensis* |
|  |  | *Garra* | *Garra imberba* |
|  |  | *Parasinilabeo* | *Parasinilabeo maculatus* |
|  |  | *Pseudogyrinocheilus* | *Pseudogyrinocheilus prochilus* |
|  |  | *Rectoris* | *Rectoris luxiensis* |
|  |  | *Sinocrossocheilus* | *Sinocrossocheilus guizhouensis* |
|  |  | *Abbottina* | *Abbottina obtusirostris* |
|  |  |  | *Abbottina rivularis* |
|  |  | *Belligobio* | *Belligobio nummifer* |
|  |  |  | *Belligobio pengxianensis* |
|  |  | *Coreius* | *Coreius guichenoti* |
|  |  |  | *Coreius heterodon* |
|  |  | *Gnathopogon* | *Gnathopogon herzensteini* |
|  |  |  | *Gnathopogon imberbis* |
|  |  |  | *Gnathopogon nicholsi* |
|  |  |  | *Gnathopogon tsinanensis* |
|  |  | *Hemibarbus* | *Hemibarbus labeo* |
|  |  |  | *Hemibarbus longirostris* |
|  |  |  | *Hemibarbus maculatus* |
|  |  | *Microphysogobio* | *Microphysogobio fukiensis* |
|  |  |  | *Microphysogobio kiatingensis* |
|  |  |  | *Microphysogobio microstomus* |
|  |  |  | *Microphysogobio tungtingensis* |
|  |  | *Paracanthobrama* | *Paracanthobrama guichenoti* |
|  |  |  | *Paracanthobrama umbrifer* |
|  |  | *Platysmacheilus* | *Platysmacheilus exiguus* |
|  |  |  | *Platysmacheilus longibarbatus* |
|  |  |  | *Platysmacheilus nudiventris* |
|  |  | *Pseudogobio* | *Pseudogobio vaillanti* |
|  |  | *Pseudorasbora* | *Pseudorasbora elongata* |
|  |  |  | *Pseudorasbora parva* |
|  |  | *Rhinogobio* | *Rhinogobio cylindricus* |
|  |  |  | *Rhinogobio hunanensis* |
|  |  |  | *Rhinogobio typus* |
|  |  |  | *Rhinogobio ventralis* |
|  |  | *Sarcocheilichthys* | *Sarcocheilichthys davidi* |
|  |  |  | *Sarcocheilichthys kiangsiensis* |
|  |  |  | *Sarcocheilichthys nigripinnis* |
|  |  |  | *Sarcocheilichthys parvus* |
|  |  |  | *Sarcocheilichthys sinensis* |
|  |  | *Saurogobio* | *Saurogobio dabryi* |
|  |  |  | *Saurogobio dumerili* |
|  |  |  | *Saurogobio gracilicaudatus* |
|  |  |  | *Saurogobio gymnocheilus* |
|  |  |  | *Saurogobio xiangjiangensis* |
|  |  | *Squalidus* | *Squalidus argentatus* |
|  |  |  | *Squalidus nitens* |
|  |  |  | *Squalidus wolterstorffi* |
|  |  | *Carassius* | *Carassius auratus* |
|  |  | *Cyprinus* | *Cyprinus carpio* |
|  |  |  | *Cyprinus chilia* |
|  |  |  | *Cyprinus micristius* |
|  |  |  | *Cyprinus qionghaiensis* |
|  |  | *Gymnocypris* | *Gymnocypris firmispinatus* |
|  |  |  | *Gymnocypris potanini* |
|  |  | *Gobiobotia* | *Gobiobotia abbreviata* |
|  |  |  | *Gobiobotia brevirostris* |
|  |  |  | *Gobiobotia filifer* |
|  |  |  | *Gobiobotia longibarba* |
|  |  |  | *Gobiobotia meridionalis* |
|  |  |  | *Gobiobotia tungi* |
|  |  | *Gobiocypris* | *Gobiocypris rarus* |
|  |  | *Xenophysogobio* | *Xenophysogobio boulengeri* |
|  |  |  | *Xenophysogobio nudicorpa* |
|  |  | *Ctenopharyngodon* | *Ctenopharyngodon idella* |
|  |  | *Mylopharyngodon* | *Mylopharyngodon piceus* |
|  |  | *Squaliobarbus* | *Squaliobarbus curriculus* |
|  |  | *Barbodes* | *Barbodes polylepis* |
|  |  | *Elopichthys* | *Elopichthys bambusa* |
|  |  | *Folifer* | *Folifer brevifilis* |
|  |  | *Gymnodiptychus* | *Gymnodiptychus pachycheilus* |
|  |  | *Hemiculterella* | *Hemiculterella sauvagei* |
|  |  |  | *Hemiculterella wui* |
|  |  |  | *Herzensteinia microcephalus* |
|  |  | *Linichthys* | *Linichthys laticeps* |
|  |  | *Luciobrama* | *Luciobrama macrocephalus* |
|  |  | *Onychostoma* | *Onychostoma angustistomata* |
|  |  |  | *Onychostoma barbatulum* |
|  |  |  | *Onychostoma barbatum* |
|  |  |  | *Onychostoma breve* |
|  |  |  | *Onychostoma elongatum* |
|  |  |  | *Onychostoma lini* |
|  |  |  | *Onychostoma macrolepis* |
|  |  |  | *Onychostoma rarum* |
|  |  |  | *Onychostoma simum* |
|  |  | *Percocypris* | *Percocypris pingi* |
|  |  | *Procypris* | *Procypris rabaudi* |
|  |  | *Ptychobarbus* | *Ptychobarbus chungtienensis* |
|  |  |  | *Ptychobarbus kaznakovi* |
|  |  | *Schizopygopsis* | *Schizopygopsis kialingensis* |
|  |  |  | *Schizopygopsis malacanthus* |
|  |  | *Sinilabeo* | *Sinilabeo longibarbatus* |
|  |  | *Spinibarbus* | *Spinibarbus hollandi* |
|  |  |  | *Spinibarbus sinensis* |
|  | Nemacheilidae | *Homatula* | *Homatula potanini* |
|  |  |  | *Homatula variegata* |
|  |  |  | *Homatula wujiangensis* |
|  |  | *Claea* | *Claea dabryi* |
|  |  | *Schistura* | *Schistura fasciolata* |
|  |  |  | *Schistura incerta* |
|  |  |  | *Schistura waltoni* |
|  |  | *Triplophysa* | *Triplophysa angeli* |
|  |  |  | *Triplophysa anterodorsalis* |
|  |  |  | *Triplophysa bleekeri* |
|  |  |  | *Triplophysa brevibarba* |
|  |  |  | *Triplophysa brevicauda* |
|  |  |  | *Triplophysa daqiaoensis* |
|  |  |  | *Triplophysa grahami* |
|  |  |  | *Triplophysa leptosoma* |
|  |  |  | *Triplophysa markehenensis* |
|  |  |  | *Triplophysa microps* |
|  |  |  | *Triplophysa ninglangensis* |
|  |  |  | *Triplophysa orientalis* |
|  |  |  | *Triplophysa rotundiventris* |
|  |  |  | *Triplophysa stenura* |
|  |  |  | *Triplophysa stewarti* |
|  |  |  | *Triplophysa stoliczkai* |
|  |  |  | *Triplophysa tanggulaensis* |
|  |  |  | *Triplophysa xichangensis* |
|  |  |  | *Triplophysa yaopeizhii* |
|  |  | *Yunnanilus* | *Yunnanilus nigromaculatus* |
|  |  |  | *Yunnanilus pleurotaenia* |
|  | Balitoridae | *Beaufortia* | *Beaufortia liui* |
|  |  |  | *Beaufortia szechuanensis* |
|  |  | *Erromyzon* | *Erromyzon sinensis* |
|  |  | *Formosania* | *Formosania davidi* |
|  |  | *Hemimyzon* | *Hemimyzon yaotanensis* |
|  |  | *Jinshaia* | *Jinshaia abbreviata* |
|  |  |  | *Jinshaia sinensis* |
|  |  |  | *Jinshaia niulanjiangensis* |
|  |  | *Lepturichthys* | *Lepturichthys fimbriata* |
|  |  | *Metahomaloptera* | *Metahomaloptera omeiensis* |
|  |  |  | *Metahomaloptera longicauda* |
|  |  | *Paraprotomyzon* | *Paraprotomyzon niulanjiangensis* |
|  |  | *Pseudogastromyzon* | *Pseudogastromyzon changtingensis* |
|  |  |  | *Pseudogastromyzon fangi* |
|  |  |  | *Pseudogastromyzon fasciatus* |
|  |  | *Sinogastromyzon* | *Sinogastromyzon hsiashiensis* |
|  |  |  | *Sinogastromyzon sichangensis* |
|  |  |  | *Sinogastromyzon szechuanensis* |
|  |  |  | *Sinogastromyzon dezeensis* |
|  |  | *Vanmanenia* | *Vanmanenia pingchowensis* |
|  |  | *Formosania* | *Formosania stigmata* |
|  |  | *Vanmanenia* | *Vanmanenia stenosoma* |
|  |  |  | *Vanmanenia xinyiensis* |
|  | Cobitidae | *Cobitis* | *Cobitis macrostigma* |
|  |  |  | *Cobitis sinensis* |
|  |  |  | *Cobitis taenia* |
|  |  |  | *Cobitis rara* |
|  |  | *Misgurnus* | *Misgurnus anguillicaudatus* |
|  |  |  | *Misgurnus mizolepis* |
|  |  | *Paramisgurnus* | *Paramisgurnus dabryanus* |
|  |  | *Leptobotia* | *Leptobotia elongata* |
|  |  |  | *Leptobotia guilinensis* |
|  |  |  | *Leptobotia microphthalma* |
|  |  |  | *Leptobotia orientalis* |
|  |  |  | *Leptobotia pellegrini* |
|  |  |  | *Leptobotia rubrilabris* |
|  |  |  | *Leptobotia taeniops* |
|  |  |  | *Leptobotia tchangi* |
|  |  |  | *Leptobotia tientaiensis* |
|  |  | *Parabotia* | *Parabotia banarescui* |
|  |  |  | *Parabotia bimaculata* |
|  |  |  | *Parabotia fasciata* |
|  |  |  | *Parabotia kiangsiensis* |
|  |  |  | *Parabotia maculosa* |
|  |  |  | *Parabotia lijiangensis* |
|  |  | *Sinibotia* | *Sinibotia reevesae* |
|  |  |  | *Sinibotia superciliaris* |
| Siluriformes | Siluridae | *Silurus* | *Silurus asotus* |
|  |  |  | *Silurus meridionalis* |
|  | Clariidae | *Clarias* | *Clarias fuscus* |
|  | Bagridae | *Hemibagrus* | *Hemibagrus macropterus* |
|  |  | *Leiocassis* | *Leiocassis crassilabris* |
|  |  |  | *Leiocassis crassirostris* |
|  |  |  | *Leiocassis longibarbus* |
|  |  |  | *Leiocassis longirostris* |
|  |  | *Pelteobagrus* | *Pelteobagrus eupogon* |
|  |  |  | *Pelteobagrus ussuriensis* |
|  |  |  | *Pseudobagrus albomarginatus* |
|  |  |  | *Pseudobagrus analis* |
|  |  |  | *Pseudobagrus brevicaudatus* |
|  |  |  | *Pseudobagrus crassilabris* |
|  |  |  | *Pseudobagrus medianalis* |
|  |  |  | *Pseudobagrus ondon* |
|  |  |  | *Pseudobagrus pratti* |
|  |  |  | *Pseudobagrus taeniatus* |
|  |  |  | *Pseudobagrus tenuifurcatus* |
|  |  |  | *Pseudobagrus tenuis* |
|  |  |  | *Pseudobagrus truncatus* |
|  |  |  | *Pseudobagrus vachellii* |
|  |  | *Tachysurus* | *Tachysurus adiposalis* |
|  |  |  | *Tachysurus fulvidraco* |
|  |  |  | *Tachysurus nitidus* |
|  | Amblycipitidae | *Liobagrus* | *Liobagrus marginatoides* |
|  |  |  | *Liobagrus marginatus* |
|  |  |  | *Liobagrus nigricauda* |
|  |  |  | *Liobagrus styani* |
|  | Sisoridae | *Euchiloglanis* | *Euchiloglanis kishinouyei* |
|  |  | *Glyptothorax* | *Glyptothorax fokiensis* |
|  |  |  | *Glyptothorax sinensis* |
|  |  | *Pareuchiloglanis* | *Pareuchiloglanis anteanalis* |
|  |  |  | *Pareuchiloglanis sinensis* |
| Aulopiformes | Synodontidae | *Harpadon* | *Harpadon nehereus* |
| Cypriodontiformes | Poeciliidae | *Gambusia* | *Gambusia affinis* |
| Beloniformes | Adrianichthyidae | *Oryzias* | *Oryzias latipes* |
|  |  |  | *Oryzias sinensis* |
|  | Hemiramphidae | *Hyporhamphus* | *Hyporhamphus intermedius* |
| Mugiliformes | Mugilidae | *Liza* | *Liza carinata* |
|  |  |  | *Liza haematocheila* |
|  |  | *Mugil* | *Mugil cephalus* |
| Synbranchiformes | Synbranchidae | *Monopterus* | *Monopterus albus* |
|  | Mastacembelidae | *Mastacembelus* | *Mastacembelus aculeatus* |
|  |  |  | *Mastacembelus armatus* |
|  |  | *Sinobdella* | *Sinobdella sinensis* |
| Perciformes | Lateolabracidae | *Lateolabrax* | *Lateolabrax japonicus* |
|  | Percichthyidae | *Coreoperca* | *Coreoperca whiteheadi* |
|  |  | *Siniperca* | *Siniperca chuatsi* |
|  |  |  | *Siniperca knerii* |
|  |  |  | *Siniperca obscura* |
|  |  |  | *Siniperca roulei* |
|  |  |  | *Siniperca scherzeri* |
|  |  |  | *Siniperca undulata* |
|  | Eleotridae | *Bostrychus* | *Bostrychus sinensis* |
|  |  | *Eleotris* | *Eleotris oxycephala* |
|  | Odontobutidae | *Micropercops* | *Micropercops swinhonis* |
|  |  | *Odontobutis* | *Odontobutis obscura* |
|  | Gobiidae | *Glossogobius* | *Glossogobius giuris* |
|  |  | *Lophiogobius* | *Lophiogobius ocellicauda* |
|  |  | *Acanthogobius* | *Acanthogobius luridus* |
|  |  | *Chaeturichthys* | *Chaeturichthys stigmatias* |
|  |  | *Ctenogobius* | *Ctenogobius chengtuensis* |
|  |  |  | *Ctenogobius shennongensis* |
|  |  |  | *Ctenogobius szechuanensis* |
|  |  | *Mugilogobius* | *Mugilogobius myxodermus* |
|  |  | *Rhinogobius* | *Rhinogobius brunneus* |
|  |  |  | *Rhinogobius cliffordpopei* |
|  |  |  | *Rhinogobius giurinus* |
|  |  |  | *Rhinogobius similis* |
|  |  | *Synechogobius* | *Synechogobius ommaturus* |
|  |  | *Tridentiger* | *Tridentiger trigonocephalus* |
|  |  | *Odontamblyopus* | *Odontamblyopus rubicundus* |
|  |  | *Periophthalmus* | *Periophthalmus novaeguineaensis* |
|  |  | *Boleophthalmus* | *Boleophthalmus pectinirostris* |
|  | Osphronemidae | *Macropodus* | *Macropodus opercularis* |
|  | Channidae | *Channa* | *Channa argus* |
|  |  |  | *Channa asiatica* |
|  |  |  | *Channa maculata* |
|  | Callionymidae | *Repomucenus* | *Repomucenus olidus* |
| Scorpaeniformes | Cottidae | *Trachidermus* | *Trachidermus fasciatus* |
| Pleuronectiformes | Cynoglossidae | *Cynoglossus* | *Cynoglossus gracilis* |
|  |  |  | *Cynoglossus trigrammus* |
| Tetraodontiformes | Tetraodontidae | *Takifugu* | *Takifugu obscurus* |
